# Selvester score predicts implantable cardioverter defibrillator shocks in patients with non‐ischemic cardiomyopathy

**DOI:** 10.1002/joa3.12571

**Published:** 2021-06-07

**Authors:** Fazıl Arısoy, Ozlem Ozcan Celebi, İlke Erbay, Omaç Tufekcioglu, Sinan Aydoğdu, Ahmet Temizhan

**Affiliations:** ^1^ Department of Cardiology Kilis State Hospital Kilis Turkey; ^2^ Department of Cardiology University of Health Science Ankara City Hospital Ankara Turkey

**Keywords:** ICD, non‐ischemic cardiomyopathy, Selvester score, shock

## Abstract

**Background:**

The implantable cardiac defibrillator is the cornerstone of prevention of sudden cardiac death in non‐ischemic cardiomyopathy. The Selvester score, which is frequently investigated in ischemic cardiomyopathy, has not been investigated in the field of non‐ischemic cardiomyopathy.

**Aim:**

The aim of this study was to evaluate the Selvester score for determining appropriate implantable cardiac defibrillator shocks in non‐ischemic cardiomyopathy patients.

**Materials and methods:**

In all, 131 non‐ischemic cardiomyopathy patients were included in the study. A simplified Selvester score was calculated from ECG data. Patients were divided into two groups according to whether they received ICD shock.

**Results:**

Of the patients, 28.2% received appropriate implantable cardiac defibrillator shock. The Selvester score was significantly higher in patients receiving appropriate shock when compared to patients with no implantable cardiac defibrillator shocks (8.8 ± 4.6 vs 7.2 ± 3.3, *P* = .040). The median QRS duration was significantly longer in patients receiving appropriate shock than in patients with no shocks (130.14 ± 35.08 ms vs 120.12 ± 20.57 ms, *P* = .045). We determined that the cutoff value for the Selvester score to predict ICD shocks was 6.5 with a sensitivity of 72.0% and a specificity of 83% (AUC = 0.717; %95 GA: 0.627‐0.807, *P* < .001).

**Conclusion:**

Selvester score was higher in patients receiving appropriate shock than in patients who did not receive any implantable cardiac defibrillator shock. From this study, the Selvester score is associated with the risk of ventricular tachycardia/ventricular fibrillation in non‐ischemic cardiomyopathy so that careful attention is necessary to manage the patients with high Selvester score.

## Graphical abstract

Selvester score was higher in patients receiving appropriate shock than in patients who did not recieve any implantable cardiac defibrillator shock. In the selection of patients undergoing implantable cardioverter defibrillator for primer prevention of sudden cardiac death with nonischemic cardiomyopathy, the Selvester score may be used in addition to left ventricular ejection fraction.

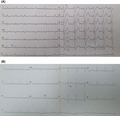

## 1 INTRODUCTION

Unlike ischemic cardiomyopathy, non‐ischemic cardiomyopathy (NICMP) consists of a heterogeneous group of diseases that affect the myocardium without significant coronary artery disease and is an important cause of sudden cardiac death.^1^, ^2^ The implantable cardioverter defibrillator (ICD) is one of the most effective interventions for the prevention of sudden cardiac death in patients with cardiomyopathy.^3^, ^4^ Both the Sudden Cardiac Death in Heart Failure Trial (SCD‐HeFT) and Multicenter Automatic Defibrillator Implantation Trial II (MADIT‐II) showed the benefits of ICD implantation in patients with cardiomyopathy.^5^, ^6^ However, Kober et al recently raised doubts about ICD implantation for the primary prevention of sudden cardiac death in patients with NICMP.^7^ This study showed that there is heterogeneity among the NICMP patients for the benefits of ICD therapy. However, the exact cause of this problem is not clear. However, there is an ongoing controversy about the patient selection criteria for ICD therapy in NICMP patients.

To date, among all NICMP types, the selection criteria of NICMP patients for primary prevention ICD implantation depend on the left ventricular ejection fraction (LVEF). However, based on the findings of Kober et al, research about selection criteria other than LVEF has become a point of interest.

The development of life‐threatening ventricular arrhythmias has been associated with myocardial scars. This relationship has been well studied in ischemic cardiomyopathy.^8^, ^9^ However, there are few data about the relationship between myocardial scars and ventricular arrhythmias in NICMP patients.^10^ It has been shown that myocardial scar tissue determined by contrast involvement in cardiac magnetic resonance imaging (MRI) increased the risk for ICD shock and cardiac death by 8‐fold in the late period in NICMP.^11^ However, imaging methods that identify scar tissue, such as cardiac MRI, are not feasible, as they require skilled technicians and are not cost‐effective. Therefore, more useful and feasible methods are needed to predict sudden cardiac death and ICD shocks in NICMP patients.

The Selvester QRS score is a scoring system created in the 1980s that provides information about the location and size of the myocardial scar, which can be determined by 12‐lead ECG.^12^ Studies have shown that the Selvester score varies between 0 and 29, and the increase in the Selvester score is directly correlated with the increase in the amount of myocardial scar tissue.^13^ The Selvester score is well studied in ischemic cardiomyopathy. However, data evaluating the value of the Selvester score in patients with NICMP are limited. Recently, the Selvester score has been reported as a determinant in the development of adverse cardiac events in NICMP patients.^14^, ^15^

In this study, we evaluated the relationship between Selvester score and ICD shocks in patients with NICMP.

## 2 METHODS

This is a cross‐sectional study. We enrolled NICMP patients with a previously implanted ICD who were admitted to our heart failure unit between March 2018 and January 2019. All included patients were at follow‐up in our department and NICMP diagnoses were made based on invasive and/or noninvasive tests including endomyocardial biopsy. Patients with ischemic cardiomyopathy, severe renal or hepatic impairment, pacemaker‐dependent or non‐diagnostic ECG findings, and ICD‐implanted patients for secondary prevention were excluded. This study was carried out with the approval of the Turkiye Yuksek Ihtisas Training and Research Hospital Clinical Research Ethics Committee (Date: 13.02.2018 and Approval No: 33) and was in accordance with the World Medical Association Helsinki Declaration.

Medical records were evaluated. Furthermore, pulmonary capillary wedge pressure (PCWP), pulmonary vascular resistance (PVR), and CI (cardiac index) measurements were obtained from the medical records of patients who underwent cardiac catheterization before study enrollment.

We included only therapies that ended with a shock. We included both VT and VF zone arrhythmias. Programming of the ICD therapy algorithms is performed according to current literature. In our department, we program the ICDs for primary prevention as VT detection zone >180 bpm with 6 ATP therapies (each for 8 beats) and VF detection zone >210 bpm with defibrillation (ATP delivery before and/or during capacitor charging) we program the ICDs for secondary prevention according to the patients’ previous VT‐VF cycle length.

Twelve‐lead ECG recordings of all patients and transthoracic echocardiography examinations were performed. All patients underwent in‐hospital ICD device monitoring. This monitorization included all data from implantation to date. Physical examination findings and biochemical and hematological laboratory measurements were also performed.

Patients were divided into two groups: the first group included patients who received at least one appropriate shock after device and the second group included patients with no ICD shock. Patients with inappropriate shocks were excluded (n = 11). Patients who received appropriate shock and did not receive shock were compared in terms of demographic features, laboratory features, cardiac features, and Selvester QRS score.

In our study, the Selvester score was used in the form simplified by Bounous et al in 1988.^16^ All ECGs were reviewed and scored by two cardiologists.

### 2.1 Statistical analysis

Statistical analysis was performed using IBM SPSS Statistics 20.0 statistical software (IBM^®^ Inc, Chicago, USA). Continuous variables were reported as the mean ± standard deviation (SD), and categorical variables were expressed as the number of patients and percentages. Normality was tested using the Kolmogorov‐Smirnov test. Comparisons between categorical variables were performed by Pearson's chi‐square test or Fisher's exact test, as appropriate. Continuous variables were compared using Student's *t*‐test for independent samples or the Mann‐Whitney test, as appropriate. Pearson and Spearman correlation tests were used in the correlation analysis. Stepwise multiple logistic regression analysis was performed to test the relationship between risk factors and appropriate shocks. Area under the (receiver operating characteristic) curve (AUC), based on C‐statistics, was performed to determine the optimal cutoff value for the Selvester score to predict the appropriate ICD shock. ROC analysis was expressed as the AUC and 95% confidence interval. A *P* value <.05 was considered statistically significant.

## 3 RESULTS

**FIGURE 1:**
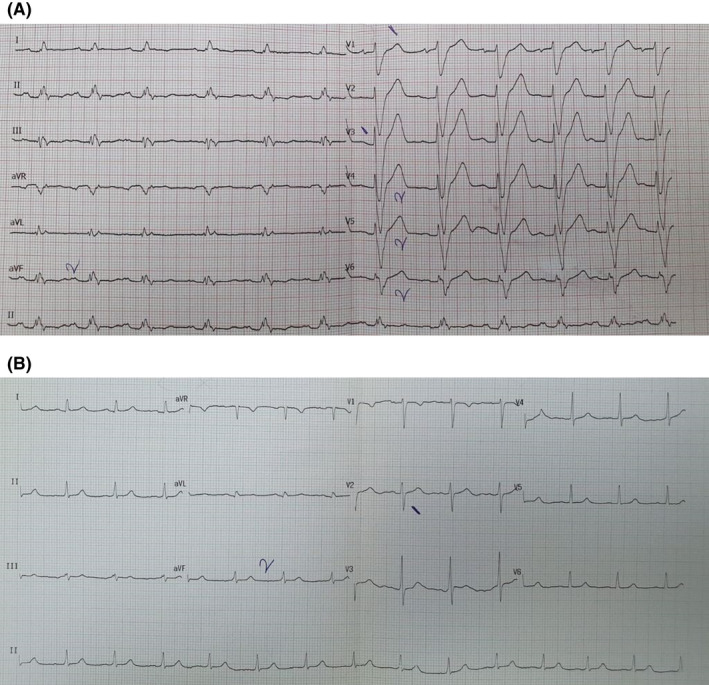
A, 12 Lead electrocardiogram of a patient who received appropriate ICD shock. B, 12 Lead electrocardiogram of a patient without any ICD shock

**FIGURE 2:**
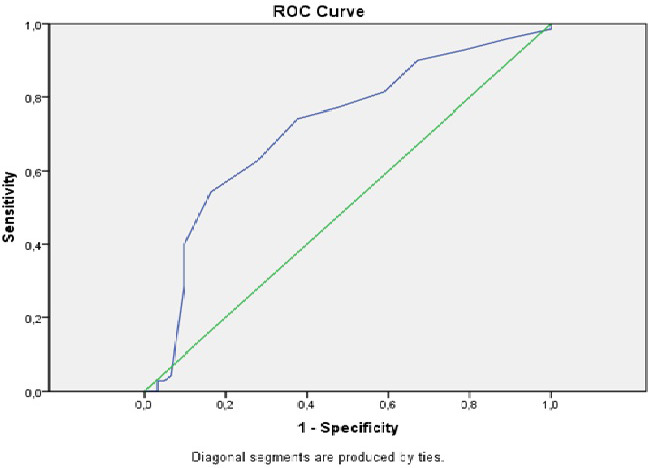
The receiver operating characteristic curves of Selvester Score for predicting appropriate implantable cardioverter defibrillator shock in patients with non‐ischemic cardiomyopathy

Between March 2018 and January 2019, a total of 131 patients were included (mean age: 49.6 ± 13.2 years and 70.2% males). Table 1 demonstrates the baseline clinical characteristics of the patients. Table 2 shows the manufacturers and modes of implanted ICDs. We determined that 37 patients (28.2%) had at least one appropriate ICD shock after the implantation of the device. Among the study patients, 83 patients (63.4%) did not have any ICD shocks, and 11 patients (8.4%) had an inappropriate ICD shock. Figure 1A and B shows the ECGs of patients with and without ICD shocks. None of the study patients developed both inappropriate and appropriate ICD shocks. Patients with appropriate ICD shock had lower LVEF and estimated glomerular filtration rate (eGFR) values than patients with no ICD shock (23.3 ± 9.5% vs 27.4 ± 8.9%, *P* = .002 and 68 ± 30.2 mL/min vs 79 ± 32.7 mL/min, *P* = .027, respectively). The QRS duration in 12‐lead ECGs was longer, and the Selvester score values were higher in patients with appropriate ICD shock than in patients with no ICD shock (130.14 ± 35.08 ms vs 120.12 ± 20.57 ms, *P* = .045 and 8.8 ± 4.6 vs 7.2 ± 3.3, *P* = .040, respectively). Additionally, the rate of the presence of left anterior hemiblock and presence of notching in any of the leads were higher in patients with appropriate ICD shock than in patients with no ICD shock (Table 3). The multiple logistic regression analysis revealed that male gender, Selvester score, and LVEF were independent predictors of appropriate ICD shock (Table 4). ROC analysis showed that the cutoff value for the Selvester score to predict ICD shocks was 6.5 with a sensitivity of 72.0% and a specificity of 83% (AUC = 0.717; %95 GA 0.627‐0.807, *P* < .001) (Figure 2).

**TABLE 1:** Basal characteristics of patients with ICD shock and without ICD shock

	Appropriate ICD shock (n = 37)	No ICD Shock (n = 83)	*P* value
Age (years)	52.8 ± 13.6	48.2 ± 12.5	.075
Male n (%)	28 (75.7)	57 (68.7)	.436
BMI (kg/m^2^)	27.5 ± 3.9	27.9 ± 4.9	.673
DM n (%)	7 (18.9)	13 (15.7)	.658
SBP (mmHg)	105 ± 25.5	112 ± 27.1	.436
DBP (mmHg)	69 ± 17.5	76 ± 16.2	. ***006***
Hospitalization* n (%)	23 (62.2)	21 (25.3)	***<.001***
Smoking n (%)	18 (48.6)	32 (38.6)	.300
Medication			
Beta‐blocker n (%)	37 (100)	81 (97.6)	.341
Amiodarone n (%)	18 (48.6)	5 (6)	***<.001***
Diuretics n (%)	27 (73)	69 (83.1)	.199
ACEI/ARB n (%)	30 (81.1)	76 (91.6)	.098
Digitalis n (%)	11 (29.7)	24 (28.9)	.928
MRA n (%)	29 (78.4)	76 (91.6)	. ***044***
NYHA			
I	6 (16.2)	12 (14.5)	
II	16 (43.2)	45 (54.2)	.523
III	15 (40.5)	26 (31.3)	
IV	0	0	
AF n (%)	10 (27)	23 (27.7)	.938
QRS duration (ms)	130 ± 35	120 ± 20	. ***045***
Selvester score	8.8 ± 4.6	7.2 ± 3.3	. ***040***
LAD (cm)	4.5 ± 1.1	4.6 ± 1.2	.264
LVEF (%)	23.3 ± 9.5	27.4 ± 8.9	. ***002***
LVEDD (cm)	6.1 ± 1.3	6.3 ± 1.1	.952
Hb (g/dL)	13.4 ± 2.3	14.3 ± 1.7	. ***026***
Htc (%)	41.0 ± 6.4	43.7 ± 4.7	. ***030***
PLT (10^3^/µL)	218 ± 90	238 ± 74	.224
PDW (%)	13.1 ± 2.2	12.8 ± 2.5	.576
MCV (fL)	87.0 ± 7.5	86.3 ± 6.9	.938
WBC (10^3^/µL)	8.8 ± 2.3	8.0 ± 1.8	.*062*
Neutrophil (%)	66.9 ± 9.0	62.2 ± 8.8	. ***009***
Lymphocyte (%)	22.3 ± 8.1	26.1 ± 7.9	. ***015***
Na (mmol/L)	138 ± 4.5	136 ± 3.7	.991
K (mmol/L)	4.4 ± 0.9	4.2 ± 0.4	.999
eGFR (mL/min/1.73 m^2^)	68 ± 30.2	79 ± 32.7	. ***027***
BNP (pg/mL), median	1388 (4520)	1058 (1994)	.225
Serum Creatinin (mg/dL)	1.36 ± 0.57	1.21 ± 0.46	. ***034***
PCWP (mmHg), Median (n = 87)	17 (12)	17 (11)	.426
PVR (dynes·sec/cm^2^), median, n (=87)	2.0 (1‐7)	2.0 (1‐6)	.501
CI (L/dk/m^2^), median, (n = 87)	2.1 (1‐4)	2.3 (1‐4)	.905

Abbreviations: ACEI, Angiotensin‐converting‐enzyme inhibitors; AF, Atrial fibrillation; ARB, Angiotensin receptor blockers; BMI, Body Mass Index; BNP, Brain natriuretic peptide; CI, Cardiac index; DBP, Diastolic blood pressure; DM, Diabetes Mellitus; eGFR, estimated glomerular filtration; Hb, Hemoglobin; Htc, hematocrit; ICD, Implantable cardioverter defibrillator; K, Potassium; LAD, Left atrial diameter; LVEDD, Left ventricle end diastolic diameter; LVEF, Left ventricle ejection fraction; MRA, Mineralocorticoid Receptor Antagonists; MVC, mean corpuscular volume; Na, Sodium; NYHA, New York Heart Association; PCWP, pulmonary capillary wedge pressure; PDW, Platelet distribution width; PLT, Platelet; PVR, Pulmonary vascular resistance; SBP, Systolic blood pressure; WBC, White blood cell rate.

Bold and italic indicate significant value (*P* < .05).

*Hospitalization during the last year.

**TABLE 2:** Manufacturers and modes of ICDs

Manufacturer	VVI, n (%)	DDD, n (%)	CRT n (%)	Total, n (%)
Medtronic	45 (67.1)	10 (14.9)	12 (17.9)	67 (51.1)
Boston Scientific	17 (53.1)	8 (25)	7 (21.8)	32 (24.4)
St. Jude Medical	16 (88.8)	2 (11.1)	0	18 (13.7)
Biotronik	6 (54.5)	1 (9.1)	4 (36.3)	11 (8.4)
Sorin‐Ela	3 (100)	0	0	3 (2.3)

Abbreviations: CRT, cardiac resynchronization therapy ICD; DDD, dual‐chamber ICD; ICD, implantable cardioverter defibrillator; VVI, single‐chamber ICD.

**TABLE 3:** ECG findings

	Appropriate ICD shocks	No ICD shock	*P* value
Heart rate (bpm)	86.1 ± 22.1	84.4 ± 17.15	.225
PR interval (ms)	0.18 ± 0.24	0.18 ± 0.22	.800
QT interval (ms)	402.60 ± 22.55	403.64 ± 19.32	.591
QRS duration (ms)	130.14 ± 35.08	120.12 ± 20.57	.045
Presence of Notchs in any leads, n (%)	12 (32.4)	20 (24.1)	.020
Presence of LAHB (%)	10 (27.1)	9 (10.8)	.010

Abbreviations: ICD, implantable cardioverter defibrillator; LAHB, left anterior hemiblock.

**TABLE 4:** Multivariate logistic regression analysis of factors related to appropriate ICD shock

	Odds ratio	95%CI	*P*
Age	0.786	0.512‐2.103	.349
Male gender	1.921	1.034‐4.792	.016
LVEF	1.865	1.726‐3.129	.038
Selvester score	1.971	1.385‐4.537	.008
BNP	0.903	0.648‐1.152	.186
Smoking	1.175	0.968‐3.496	.059

Abbreviations: BNP, Brain natriuretic peptideI; CD, Implantable cardioverter defibrillator; LVEF, Left ventricle ejection fraction.

## 4 DISCUSSION

In our study, we determined that the Selvester score was higher in those who received appropriate ICD shock than in those without ICD shock. Additionally, the Selvester score was a predictor of appropriate ICD shocks and the cutoff value of the Selvester score to predict appropriate ICD shock was 6.5.

ICD, which has been shown by many studies to decrease sudden cardiac death, is widely used in clinical practice.^17^ Current European Society of Cardiology (ESC) guidelines recommend ICD implantation in primary prevention of sudden cardiac death (without emergence of sudden cardiac death) in patients with NICMP with NYHA class II and III and LVEF <35% (Class I, level of evidence B).^18^ Although LVEF is considered the major determinant in ICD implantation, it shows low sensitivity and specificity for sudden cardiac death.^19^, ^20^ After the study by Køber et al, ICD implantation indications have been opened for discussion, resulting in the belief that new criteria may be required for the selection of patients for implantation.^7^ There are data in the literature that myocardial scars may be important in determining the ICD indication in patients with ischemic cardiomyopathy.^21^ It is known that the Selvester score can show myocardial scars even in the presence of abnormal ventricular conduction. However, data on this issue are limited in patients who have ischemic cardiomyopathy. Myocardial scarring and fibrosis are the triggers of arrhythmias not only in ischemic cardiomyopathy but also in NICMP, but limited data are present.^22^ Our study showed that the Selvester score, a predictor of myocardial scarring, may be related to ventricular arrhythmias in patients with NICMP. Similarly, in their recent study, Hiraiwa et al reported that the Selvester score is an independent determinant of adverse cardiac events in patients with NICMP.^14^ In the cardiac MRI study carried out by Wu et al, it was reported that myocardial scar tissue increased the frequency of ICD shock in NICMP.^11^ The study conducted by Safak et al stated that the presence of inflammation was decisive in endomyocardial biopsy in idiopathic cardiomyopathy patients in appropriate ICD shocks.^23^ Also it has been showed that inflammation, and myocardial stretch, is associated with myocardial damage and fibrosis which predispose arrhythmias. Scott et al determined that biomarkers of inflammation are associated with appropriate ICD therapies.^24^ For this reason, the Selvester score, which is an inexpensive, noninvasive, easily accessible and applicable method and correlates with myocardial fibrosis, scarring may be decisive in appropriate ICD shocks in NICMP patients. To the best of our knowledge, no other study has evaluated the Selvester score in predicting ICD shocks in NICMP patients.

In NICMP, QRS duration, QT interval, QT dispersion, heart rate variability, baroreflex sensitivity, microvolt T‐wave change test, and delayed contrast involvement (myocardial scar tissue) in cardiac magnetic resonance imaging (MRI) have been examined in the selection of patients to be treated with ICD thus far. However, the best candidates were stated to have a microvolt T‐wave change test and delayed contrast involvement in cardiac MRI.^19^ Recently, the predictivity of a different QRS scoring system that is not affected by ECG confounders has been evaluated in non‐ischemic and ischemic cardiomyopathy patients.^15^ In their study, Strauss et al evaluated the effectiveness of a different QRS scoring system in determining appropriate ICD shocks in predicting ICD shocks in NYHA class II and III ischemic and NICMP patients whose LVEF was below 35%. The study reported that ischemic etiology is determinant in ICD shock and that non‐ischemic etiology was missing. When all the patients were considered together, the absence of myocardial scars according to the QRS scoring system decreased ICD shock frequency. However, it should be noted that the scoring system used in the Strauss study was different from the scoring system used in our study and that both patients with cardiomyopathy were evaluated.

We observed that the patients who received appropriate shock had different clinical and cardiac characteristics than those who did not receive a shock. In patients receiving appropriate shock, diastolic blood pressure was significantly higher, hospitalization frequency was higher, amiodarone usage was more common, the use of spironolactone was less common, and QRS duration was longer. In addition, hemoglobin and hematocrit levels were significantly lower; in patients receiving appropriate shock, the neutrophil rate was higher, the lymphocyte ratio was lower, and the GFR was even lower.

We determined that 28.2% of our patients received appropriate ICD shock, 8.4% received inappropriate ICD shock, and 63.4% did not receive any shock. Our results were consistent with the literature data. In the study conducted by Poole et al, 22.4% of patients who were subjected to ICD received appropriate shock, and 10.7% received inappropriate shock. Desai et al reported that 30% of 549 patients received appropriate shock and 13% received inappropriate shock.^25^

### 4.1 Limitations

Our study had some limitations. First, the number of patients was relatively limited. However, the number of patients was decreased due to the comprehensive exclusion criteria and that ECG confounders were not included in the study. Second, the time from implantation to enrollment differs among study patients, and this study is not a follow‐up study. Third, we did not include all patients who were candidates for ICD therapy, we included patients with an ICD. Finally, we did not evaluate the association between the number of ICD shock and Selvester score. Therefore, our findings should be interpreted carefully.

## 5 CONCLUSION

In conclusion, Selvester score was related with the incidence of ventricular arrhythmia in ICD patients. Further studies are required for evaluating the possible usefulness of Selvester score for selecting ICD candidates.

## CONFLICT OF INTEREST

None.
